# Graphene nanocomposites for real-time electrochemical sensing of nitric oxide in biological systems

**DOI:** 10.1063/5.0162640

**Published:** 2023-11-30

**Authors:** Tanveer A. Tabish, Yangzhi Zhu, Shubhangi Shukla, Sachin Kadian, Gurneet S. Sangha, Craig A. Lygate, Roger J. Narayan

**Affiliations:** 1 Division of Cardiovascular Medicine, Radcliffe Department of Medicine, British Heart Foundation (BHF) Centre of Research Excellence, University of Oxford, Oxford OX3 7BN, United Kingdom; 2 Terasaki Institute for Biomedical Innovation, Los Angeles, California 90064, USA; 3 Joint Department of Biomedical Engineering, University of North Carolina and North Carolina State University, Raleigh, North Carolina 27695-7907, USA; 4 Fischell Department of Bioengineering, University of Maryland, 8278 Paint Branch Dr., College Park, Maryland 20742, USA

## Abstract

Nitric oxide (NO) signaling plays many pivotal roles impacting almost every organ function in mammalian physiology, most notably in cardiovascular homeostasis, inflammation, and neurological regulation. Consequently, the ability to make real-time and continuous measurements of NO is a prerequisite research tool to understand fundamental biology in health and disease. Despite considerable success in the electrochemical sensing of NO, challenges remain to optimize rapid and highly sensitive detection, without interference from other species, in both cultured cells and *in vivo*. Achieving these goals depends on the choice of electrode material and the electrode surface modification, with graphene nanostructures recently reported to enhance the electrocatalytic detection of NO. Due to its single-atom thickness, high specific surface area, and highest electron mobility, graphene holds promise for electrochemical sensing of NO with unprecedented sensitivity and specificity even at sub-nanomolar concentrations. The non-covalent functionalization of graphene through supermolecular interactions, including π–π stacking and electrostatic interaction, facilitates the successful immobilization of other high electrolytic materials and heme biomolecules on graphene while maintaining the structural integrity and morphology of graphene sheets. Such nanocomposites have been optimized for the highly sensitive and specific detection of NO under physiologically relevant conditions. In this review, we examine the building blocks of these graphene-based electrochemical sensors, including the conjugation of different electrolytic materials and biomolecules on graphene, and sensing mechanisms, by reflecting on the recent developments in materials and engineering for real-time detection of NO in biological systems.

## I Introduction

Nitric oxide (NO) was first identified as an important biomolecule in the 1980s, when it was confirmed as the elusive endothelium-derived relaxing factor (EDRF);^1^ it is constitutively produced by vascular endothelial cells to regulate blood vessel tone, thereby helping to maintain normal blood pressure. It is generated endogenously in mammalian cells by a family of three nitric oxide synthase (NOS) isoforms using L-arginine and oxygen as substrates, alongside multiple essential co-factors. An impairment in NO bioavailability contributes to many cardiovascular diseases (e.g., atherosclerosis, hypertension, and heart failure^2–4^), but it is also highly important in other pathologies, such as erectile dysfunction and sepsis.^5–9^

NO is a free radical species that is highly reactive, particularly with oxygen, superoxide, and metals. Three redox-related and chemically distinct nitrogen centered species are nitrosonium cation (NO^+^), NO, and nitroxyl anion (NO^−^) with nitrogen oxidation numbers of +3, +2, and +1, respectively. Concentrations of NO reported *in vivo* vary widely and depend on the biological source, tissue oxygen levels, and NO scavenging/consumption, so while levels in the micromolar range have been reported, physiologically relevant levels may be as low as 0.1 to 5nM.^10^ NO is highly diffusible with an approximate diffusion rate of 50 *μ*m/s in media,^11^ but scavenging and inactivation by oxygen-centered species and heme-containing proteins restrict its half-life in biological systems to less than 5s. For example, NO is rapidly scavenged in blood due to an interaction with the highly abundant ferrous protein oxyhemoglobin [Hb(II)-O_2_]. In the presence of O_2_, ferrous heme proteins catalyze the NO-dioxygenase reaction, whereby NO is oxidized to nitrate (NO_3_^−^) and ferric methemoglobin [met-Hb(III)] via peroxynitrite [N(O)OO^−^] formation (shown in the following equation):^12–14^

(1) $$\text{Hb}(\text{II})-O_{2}+\text{NO}\to \text{Hb}(\text{III})N(O){\text{OO}}^{-}\to \;\text{met}\;-\text{Hb}(\text{II})+{\text{NO}}_{3}^{-}.$$

This type of reaction is also central to the physiological effects of NO via direct binding and activation of the ferrous protein soluble guanylate cyclase (sGC) to initiate downstream signaling cascades. This creates a challenge for precise real-time measurements. Ideally, the detection method needs to be in close proximity to the source of NO; the direct detection of NO must allow for the presence of other interfering species [e.g., nitrite, nitrate, dopamine (DA), ascorbate, and L-arginine], which adversely affect consistency and reproducibility. Consequently, it is difficult to investigate real-time NO concentrations in biological systems with high spatial and temporal resolutions. In this review, we will first examine the common methods of NO detection, then explain the underlying principles of electrochemical detection, before reviewing in detail the potential for graphene to improve these detection systems.

## II Measurement of NO in Biological Samples

Several spectroscopic and electrochemical methods have been used to determine NO concentrations in biological systems. Spectroscopic methods are based on both indirect and direct methods. The Griess reaction and chemiluminescence involve the indirect quantification of by-products of reactions between NO and other species, while absorbance, fluorescence, and electron paramagnetic resonance (EPR) provide the direct measurement of adducts produced between NO and complexes, e.g., fluorescent dyes, and spin traps, respectively.^15–17^

One of the simplest and most commonly used methods to indirectly measure NO is the Griess assay, which involves the spectrophotometric determination of its stable decomposition products, specifically nitrite (NO_2_^−^), but also nitrate (NO_3_^−^) following chemical reduction to nitrite. The assay is based on the Griess reaction, whereby nitrite under acidic conditions reacts with sulfanilamide to generate a diazonium ion which then combines with *N*-(1-napthyl)ethylenediamine to form an azo dye that can be detected spectrophotometrically at 540 nm.^18^ This allows for the quantification of nitrites in deproteinized biological fluids but is not very sensitive [limit of detection ~0.5 *μ*M]^19^

Detection of NO using fluorescent probes, such as diaminofluoresceins (DAFs), can uniquely provide spatial and temporal information; the use of membrane-permeable formulations allows for intracellular imaging. These probes typically exhibit high sensitivity^20^ (e.g., a detection limit in the region of 5 nM) and can be used with standard green fluorescence detection systems (e.g., microscopy, flow cytometry, or micro-plate reader). Although their predominant use is in cell culture, they have also been used for intra-vital microscopy and in *ex vivo* tissues Two different mechanisms have been proposed for the detection of NO by DAFs. The first is via autooxidation of NO in the presence of molecular oxygen to form NO_2_ or N_2_O_3_, which results in nitrosation of the diamino group to form a fluorescent triazole. The second mechanism is the oxidation of DAF to form a radical intermediate which then reacts with NO to form the triazole.^21^ The limitations of fluorescent dyes are the potential for interference from other biological molecules, the generation of NO intermediates such as N_2_O_3_, as well as the requirement for aerobic conditions to react with NO, which limits use in hypoxic conditions,^19^ a feature of several relevant diseases states (e.g., ischemia, solid tumors, and wound healing).

Chemiluminescence is one of the most sensitive and reproducible methods to detect NO via metabolites such as nitrate and nitrite. These metabolites are reduced back to NO, which then reacts with ozone to form nitrogen dioxide in an excited state. When it returns to the ground state, a photon is emitted and detected by a photomultiplier tube. Both gas and liquid phase samples, including cell lysates and tissue homogenates, can be used for this technique. The limitations of ozone chemiluminescence include expensive instrumentation, limited specificity, and sensitivity to environmental and biological conditions such as solvent type, pH, and temperature, which, in turn, affect the rate of chemiluminescent reaction.^15,22^

In comparison, EPR is a method for studying spin-active materials with one or more unpaired electrons. EPR spin traps identify the transitions of unpaired electrons in an applied magnetic field; in this case, the unpaired electron of NO resulting from the reaction of NO with a spin-trap, which forms a more stable paramagnetic species. EPR spectroscopy offers the advantages of real-time measurement of NO in living cells/tissues with high specificity but with a detection limit of around 500 nM. The limitations of EPR spin traps are their ease of oxidation under aerobic conditions in order to form heme complexes and reactive oxygen species, which could produce NO from endogenous nitroxyl, s-nitrothiols, and nitrite.^22^

Although these methods provide chemically relevant detection information, they largely either suffer from low sensitivity, complex sample preparation, and miniaturization drawbacks, as well as interference with other NO metabolites resulting from the reduction–oxidation processes of NO in biological systems. The advantages and limitations of these methods have been summarized in Table I. However, electrochemical methods of NO sensing are superior to non-electrochemical techniques due to several advantages, such as high sensitivity, low detection limits, excellent selectivity, linearity, rapid response time, large dynamic range, low cost, ease of operation, small size, and simplified sample preparation. Electrochemical sensors can be designed to measure NO in homogenates or biological fluids and in cell culture (either supernatant or for cells cultured on the sensor) and will be the focus for the rest of this review.

## III Basic Principles of Electrochemical NO Sensors

Electrochemical sensing technology represents a significant step toward achieving real-time NO monitoring in biological systems with high sensitivity and selectivity.^29,30^ Electrochemical sensors detect an analyte of interest via oxidizing or reducing the analyte on the surface of a working electrode, which produces a signal proportional to the concentration of the analyte. Signal detection can be via amperometric^31,32^ or voltammetric^33^ methods. In amperometry, a two-electrode mode system is used where the voltage is used between the reference and working electrode to measure the current produced. This utilizes a fixed working electrode voltage against the reference electrode, and the current formed by NO oxidation is measured.^34^ In voltammetric methods, a three-electrode system is used, utilizing a varying voltage, which is applied as a function of time to measure the current produced.^35^ Amperometric methods are generally preferred over voltammetric methods in order to minimize the stray capacitance effect and because they offer rapid testing, quick response time, sensitivity, and magnitude of the signal, as well as lower detection limits.^36,37^ For these reasons, the two-electrode amperometric method has been widely used to detect NO.^37^

In an electrochemical sensor, NO is detected via either electro-reduction or electro-oxidation. Previous reviews have extensively discussed the mechanisms,^15,27,34^ but in brief, when positive (electro-oxidation) or negative (electro-reduction) potential is applied at the electrode, electroactive species are either oxidized or reduced at the electrode surface, with the current generated proportional to the concentration of NO.^15^

When there is a negative potential at the electrode, then electroreduction of NO occurs:

(2) $$2\text{NO}+2e^{-}\to N_{2}O_{2}^{2-}.$$

Electro-reduction based NO sensing is generally limited by oxygen interference. To improve the sensitivity and selectivity of electroreduction based NO detection, electrodes have been coated with transition metal complexes^38^ and metalloproteins such as hemoglobin^39^ to catalyze the reduction of NO.

However, electrochemical detection of NO is most commonly measured by its direct electro-oxidation, which involves electron transfer to form a nitrosonium cation (NO^+^) [(Eq. 3)]. In the presence of hydroxide, this forms nitrite (HNO_2_) [(Eq. 4)], and if the electrode has positive potential, then HNO_2_ may be oxidized to nitrate (NO_3_^−^) [(Eq. 5)]

(3) $$\text{NO}\to {\text{NO}}^{+}+e^{-},$$

(4) $${\text{NO}}^{+}+{\text{OH}}^{-}\to {\text{HNO}}_{2},$$

(5) $${\text{HNO}}_{2}+H_{2}O\to {\text{NO}}_{3}^{-}+2e^{-}+3H^{+}.$$

These reactions clearly highlight the potential for endogenous nitrite to act as an interfering species, and one that is typically present at higher concentrations than NO itself. Other relatively high abundance interferents include ascorbic acid (AA), ammonium, hydrogen peroxide, carbon monoxide, and uric acid (UA), among others.^15^ For this reason, the selectivity of electro-oxidative NO sensing relies heavily on the use of permselective membranes to selectively allow the passage of NO to the electrode surface. Such selectivity can be achieved via the use of membranes that are only permeable to small molecular weight gases, or by electrostatic charge repulsion to differentiate between positive and negative ions, or hydrophobic polymers that greatly increase the selectivity for NO.^19,40^ Combinations of these approaches may be used to enhance selectivity, albeit with a trade-off in reduced sensitivity. Permselective membranes may also be functionalized, for example, a carbon fiber-based electrode has been reported with a Ni–porphyrin/Nafion^®^ membrane containing ascorbic acid oxidase, which transforms ascorbic acid into the non-electroactive species dehydroascorbate.^41^

**Fig. 1:**
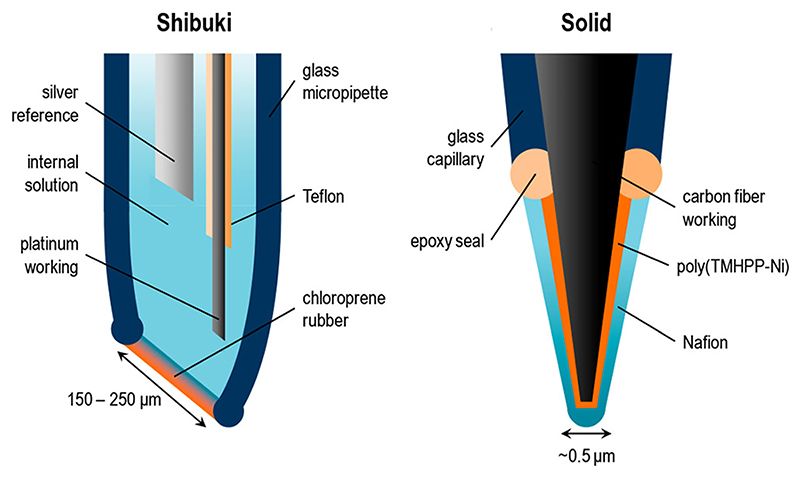
Representative schematics of Shibuki- and solid-type electrochemical NO sensors. A Shibuki-style sensor uses silver and platinum as a reference and working electrode, respectively, whereas in the solid sensor, carbon fiber acts as the working electrode with an internal or external reference electrode of silver or silver chloride. A film of oxidized TMHPP-Ni [tetrakis(3-methoxy-4-hydroxyphenyl)-nickel porphyrin] on the surface of the carbon fiber improves the sensitivity toward NO. The use of Nafion^®^ on the electrode avoids electrode fouling^43^ and is selectively permeable to NO, excluding interference from other molecules such as ascorbic acid and nitrite. Reproduced with permission from Brown and Schoenfisch, Chem. Rev. **119**, 11551–11575 (2019). Copyright 2019 American Chemical Society.^15^

In 1990, Shibuki^11^ reported the first NO electrochemical sensor based on amperometry and utilizing a modified Clark electrode to detect NO release in brain tissue. This sensor contained platinum and silver as working and reference electrodes, respectively, sitting within a glass pipette filled with electrolyte solution and the openings sealed by a gas-permeable membrane of chloroprene rubber (Fig. 1). NO generation was detected in a slice of rat cerebellum at a range of 8–58 nM. The probe was found to be selective toward NO over nitrite but with unstable sensitivity over time, ranging from 3.5–106 pA/*μ*M. The limited sensitivity is ascribed to variations in membrane thickness, permeability, and mechanical flexibility. The response time of these probes was slow, limiting utility for real-time detection of NO. Solid sensors have since been developed to eradicate the need to refill the internal electrolyte solution, utilizing a solid electrode modified with a hydrophobic membrane that is selectively permeable to the targeted molecule in order to improve selectivity and sensitivity.^19,42^ The comparison of the Shibuki NO sensor and a solid sensor is presented in Fig. 1.

In 1992, World Precision Instruments (WPI) designed the first commercial electrochemical NO sensor (ISO-NOP) following the Clarke design^44^ and has since been used extensively in research labs worldwide. This sensor comprises platinum and Ag/AgCl as the working electrode and reference electrode, respectively, covered by a stainless-steel sleeve which includes an electrolyte solution. The tip opening is positioned on the steel sleeve and is covered with a selectively permeable NO membrane. To accurately measure NO levels requires careful calibration under similar environmental conditions to the experiment (i.e., matched for the pH and temperature of the electrolyte solution) and ambient light. Calibration is performed by the construction of standard curves, which can be achieved in different ways, for example, by using NO gas directly or by decomposition of NO sources such as S-nitroso-N-acetyl-D,L-penicillamine (SNAP) in the presence of Cu (I) as a catalyst, and the reduction of nitrite into NO in the presence of potassium iodide in sulfuric acid.^45^ However, caution is required since the use of strong acids could damage the delicate membranes of NO sensors.^46^ These calibration methods have also been discussed extensively in the literature.^42,45–48^

Despite the sensitive NO detection, the major limitations of these ISI-NOP NO sensors include high noise at low NO levels, uneven background current, constant variations in the baseline, lengthy electrode polarization procedures, and requirements of frequent calibration (e.g., every 1 to 2 samples), electrode pretreatment requirements, electrode biofouling in samples (e.g., blood and NO-releasing materials), sensor degradation under harsh conditions as well as variations in electrode brittleness and size, affecting the fast detection of NO from the sample. To further improve the commercially available electrochemical NO sensors, these problems need to be addressed by developing new electrodes or modifying existing electrodes.

The sensitivity of an electrochemical sensor heavily relies on the surface area of the electrode since this enhances active and accessible sites to increase the concentration of NO on the electrode surface.^49–51^ The electrode potential is also highly important since it is a measure of the driving force for the redox reaction. High potential implies strong reduction affinity, while low potential indicates high oxidation.

The most commonly used electrodes are glassy carbon,^52,53^ gold,^54–56^ platinum,^57^ silver,^58^ and carbon fibers;^59–61^ however, most of these materials have limited sensitivity and specificity due to their limited potential to drive electrochemical oxidation reactions. The distribution of potential over the electrode surface directly influences the detection kinetics, sensitivity, and specificity. Slow electron transfer kinetics of conventional electrodes is considered a major critical issue due to their low sensitivity, which facilitates the potential required to initiate the oxidation of NO. Over the past two decades, a wide variety of nanomaterials, most notably gold nanoparticles (AuNPs),^62^ silicon nanowires (SiNWs),^63^ carbon nanotubes (CNTs),^64^ and graphene^65^ with tunable physicochemical features have been investigated as electrodes in order to improve NO sensitivity. Such nanomaterials have unique structures with highly favorable physiochemical and electrical properties^66^ and offer advantages over conventional electrode materials in terms of stability, tunable potential, and high resistance to corrosion in a wide variety of electrolytes.

Carbon-based materials have recently gained attention for electrode development in electrochemical biosensors.^67^ Among these, graphene, particularly in its functionalized form, is appealing due to its exceptional electrical, catalytical, chemical, and mechanical properties and high specific surface area. Specifically, graphene offers several advantages in terms of electrochemical sensing (e.g., high conductivity, electron mobility, and free surface energy), alongside improved current response.^51,68–70^ Several review articles have discussed nanomaterials for electrochemical sensing of NO; however, most have focused on non-graphene-based nanomaterials.^15,24,71–73^ Herein, we provide a comprehensive overview and critical evaluation of current advances in graphene-based electrochemical probes for NO detection in biological systems. The challenges in developing graphene-based NO nanoprobes, the aspects that affect performance, and the outlook of the field are also discussed.

## IV Graphene-Based Electrochemical Sensors

Graphene is a sp^2^ hybridized form of carbon atoms arranged in a repeated manner with a large surface area (2630 m^2^ g^−1^). Pristine graphene may not be an ideal candidate for electrocatalytic reactions, but it can provide a suitable surface for uniform dispersion of other active materials, thereby enhancing accessible electroactive centers for electrochemical conversions. Key features of graphene-based catalysts are large geometrical surface area, high electrical conduction, chemical stability, fast reaction kinetics, and maintenance of structural integrity under different reaction conditions. Hence, the graphene-supported nanomaterials have recently emerged as potential electrocatalysts for real-time sensing of multiple biomarkers such as dopamine, glucose, nicotinamide adenine dinucleotide, epinephrine, adenosine diphosphate (ADP), adenosine, inosine monophosphate, inosine hypoxanthine, xanthine, uric acid, and azaguanine.^74–76^

**Fig. 2:**
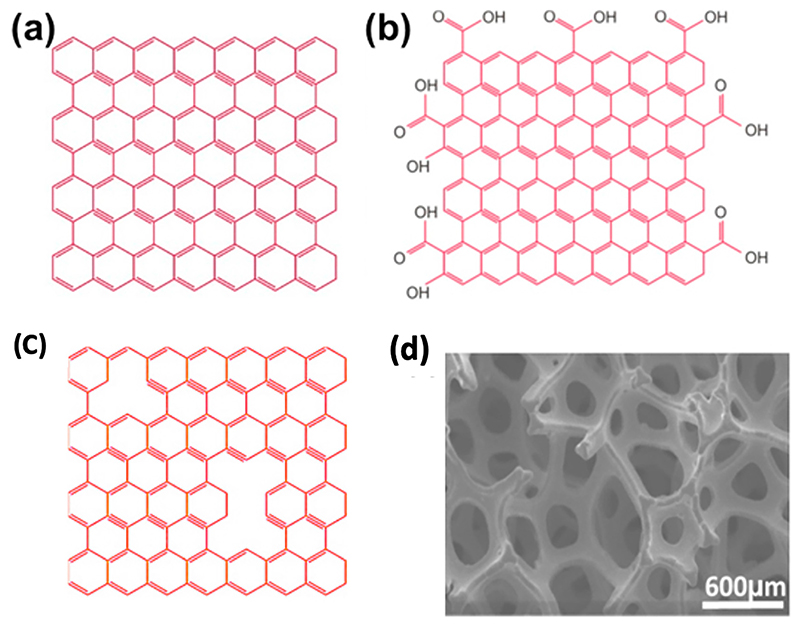
Summary of structural models of various derivatives of graphene. (a) Graphene, which is a single layer sp^2^ hybridized carbon sheet packed in a hexagonal lattice structure. Graphene has excellent properties including but not limited to high charge mobility, high specific surface area, excellent optical, electrical and mechanical characteristics and superior conductivity. (b) Graphene oxide (GO) is an oxidized form of graphene synthesized via chemical exfoliation of graphite which possesses a large number of oxygen-centered functional groups including oxygen-containing groups such as hydroxyl, epoxide, carbonyl, and carboxylic groups on its surface. The existence of functional groups offers advantages such as high chemical reactivity and hydrophilicity. (c) Reduced graphene oxide (rGO) is a reduced form of GO which has defects on its edges and basal planes. The reduction of functional groups onto the surface of GO introduces defects and pores within the graphene network. rGO shows high adsorption capacity toward different molecules due to its low oxygen content, hydrophobicity, high surface area and defects. And (d) three-dimensional (3D) graphene foam is highly porous network of graphene arranged in a micro size sheets. 3D graphene networks are found in different forms such as foam, hydrogel, aerogel etc. they offer superior porosity, specific surface area, and electrical conductivity in comparison to GO and rGO. Reproduced with permission from Tabish *et al*., Redox Biol. **15**, 34–40 (2018). Copyright 2018 Author(s), licensed under a Creative Commons Attribution (CC BY) license.^68^

Chemically modified derivatives of graphene include graphene oxide (GO),^77^ reduced graphene oxide,^78^ and three-dimensional (3D) graphene foam/hydrogels^79^ (Fig. 2). GO has functional groups of oxygen and hydrogen on the edges of graphene, which reduces conductivity due to the disruption of the sp^2^ bonding network; the conductivity of graphene is 1 × 10^8^ S m^−1^, while that of GO is about 0.02–0.07 S m^−1^.^80,81^ Through the reduction of GO, its conductivity can be regained by restoring the sp^2^ network to over 3000 S cm^−1^.^82^ Another approach to improve the conductivity of graphene is to dope it with nitrogen,^83,84^ sulfur,^85,86^ metal or metal oxide nanoparticles,^87^ or conducting polymers.^88–90^ These hybrid materials have shown superior electrochemical performance to existing metal electrodes due to the increase in charge carrier ability (i.e., the ability of a material to donate or accept electrons). The exceptionally high charge carrier mobility of graphene facilitates electron transport to rapidly accelerate the sensing response.

The conjugation and functionalization of graphene-based electrodes can be covalent or non-covalent. For example, the covalent attachment of carboxyl groups in GO with amides allows the conjugation of different elements to GO,^91^ but at the expense of lower conductivity; hence, non-covalent approaches are considered more favorable. Non-covalent functionalization involves the binding of molecules via supermolecular interactions, including π–π stacking and electrostatic interaction.^92^ Non-covalent modification of graphene does not have any effect on its structural properties, thereby preserving inherent properties. Noble metals [such as gold (Au), silver (Ag), platinum (Pt)], metal oxides, biomolecules (hemoglobin, cytochromes), have some excellent electrocatalytic properties and graphene in combination with these nanostructures has been used to further optimize electrochemical sensing of NO.

These graphene nanostructures offer multiple potential advantages due to their tunable properties, such as small size, high surface area-to-volume ratio, density of reactive sites on edges, improved electron transfer kinetics at variable potentials (by modifying their size and shape), increased NO signal and anodic peak features even at low potentials (to improve selectivity for NO oxidation), and their ability to resist degradation and fouling. Below we review studies that have made use of graphene materials for electrochemical NO sensing, and we have characterized them in terms of composite type and functionalization with respect to electro-oxidation (with metallic NPs and transition metal dichalcogenides) and electro-reduction (heme).

## V NO Detection By Graphene Nanocomposites

### A Graphene—Noble metal composites

Considerable efforts have been made to improve the morphological and surface characteristics of working electrodes for real-time monitoring of NO. The functionalization of graphene with bimetallic nanoparticles (NPs) has also emerged to significantly enhance the catalytic properties of the composite electrode. Table II compares the performances of NO sensors based on different graphene nanocomposites. An early study by Zan *et al*.^93^ reported on the development of a highly flexible hybrid electrochemical biosensor, which used graphene oxide paper coated with a bimetallic noble metal core–shell nanoparticle of Au and Pt for the monitoring of NO secreted from living cells. Core–shell particles comprise an inner core and an outer shell. The core and shell are made of different materials, and they are generally combined to achieve desired features. Intact graphene paper was obtained via a mold-casting method by taking GO dispersions produced by Hummer’s method, followed by reduction of GO (rGO) by dipping the paper in hydrogen iodide solution and drying. Later, sequentially synthesized core–shell Au@Pt NPs were exposed to ligand exchange with 2,2’-dithiobis [1-(2-bromo-2-methyl-propionyloxy)] ethane (DTBE) to create a metallic film at the oil–water interface, which was loaded on to graphene paper via dip coating. The electrochemical activity of the Au@Pt–rGO electrode was superior for the electro-oxidation of NO compared to unmodified rGO and Au–rGO electrodes, as demonstrated by reduced potential. Responses from living cells in culture were recorded by the direct growth of human umbilical vein endothelial cells (HUVECs) on top of the modified graphene paper electrodes. This flexible electrode system demonstrated high sensitivity, a wide linear range from 400 nM to 673.9 *μ*M, and a low detection limit of 100 nM for the amperometric detection of NO. In another study using graphene-bimetallic nanocomposite for NO detection, Liu *et al*.^94^ used an Au@Pt–rGO nanocomposite-modified electrochemical sensor with a linear range of 0.02–1.8 *μ*M, sensitivity of 7.35 *μ*A *μ*M^−1^, and a limit of detection of 2.88 nM.

To test the NO detection capabilities of single metallic NPs conjugated with graphene, Ting *et al*.^95^ reported the fabrication of a hybrid film of an electrochemically reduced GO (ERGO) conjugated with AuNPs. Fabrication was via a three-step process. Exfoliated sheets of GO were loaded electrophoretically onto a glassy carbon electrode (GCE) by dip coating due to the electrostatic attraction between the negatively charged GO sheets and the positively biased electrode. The gold NPs were grown electrochemically on reduced GO to obtain ERGO-AuNPs@GCE. Similarly, GCE–ERGO and GCE–ERGO modified GCEs were made to compare the reactivity of functionalized and unfunctionalized electrodes. The ERGO@AuNPs electrode proved to have superior electrocatalytic activity due to the presence of highly conductive pathways and a large surface area which facilitated electron conduction and catalysis, while the AuNPs acted as highly catalytic centers for sub-micromolar NO sensing. This synergistic combination resulted in a fast response time of 3 s and a high sensitivity (5.38 *μ*A/*μ*M/cm^2^, low detection limit: 133 nM with a S/N = ~5.5) for the selective electrochemical detection of NO in living cultured cells. In another study, Yusoff *et al*.^96^ applied a one-step hydrothermal process to develop a reduced GO and Nafion^®^ (Nf) based film loaded with nearly spherical AuNP (rGO-Nf@Au) with average diameters of ~50 to 200 nm and was cast onto the surface of a GCE. Sodium nitrite (NaNO_2_) was used as NO source, which undergoes a disproportionation reaction to release free NO in an acidic medium. The highest anodic peak current in the rGO-Nf@Au modified electrode showed the highest electro-oxidation of NO. Ikhsan *et al*.^58^ investigated the electrochemical properties of Ag NPs-supported rGO nanocomposite; they modified the GCE with prepared nanocomposite and used the modified electrode for NO oxidation experiments. This electrode exhibited a higher catalytic response as compared to bare electrodes in cyclic voltammetry for the oxidation of NO with high sensitivity, low detection limit, and excellent selectivity in the presence of common interferences. Similarly, Wang *et al*.^97^ used a nanocomposite of graphene, Nafion^®^, and gold NPs to modify the GCE for the determination of NO. Initially, the gold NPs were deposited on the GCE, and then a film of graphene was coated on it, followed by Nafion^®^ functionalization. Upon modification of GCE, the cyclic voltammetry and amperometry results showed a negative shift in the anodic peak potential (by 220 mV) which is advantageous for easy electrochemical oxidation of NO. The analytical results indicated that the developed sensor was highly sensitive to NO with linear response ranging from 36 nM to 20 *μ*M; a detection limit of 18 nM was noted. Furthermore, the developed sensor exhibited good sensitivity for NO secreted from fish liver homogenate stimulated by L-arginine.

Gold nanorods (AuNRs) conjugated with graphene have also been applied for electrochemical sensing.^98^ The interest in AuNRs arises from their elongated structures, which imbue unique optical and electronic properties. For example, Jayabal *et al*.^99^ fabricated an electrochemical NO sensor based on an amine-functionalized silicatesol–gel matrix loaded with rGO, *N*^1^-[3-(trimethoxysilyl)propyl]diethylene triamine (TPDT) and AuNR composite (rGO–Au–TPDT NRs). Comparing differently modified GCEs (bare GCE, GCE–Au–TPDT NRs, and GCE–rGO–Au–TPDT NRs), it was the GCE–rGO–Au–TPDT NRs electrode that demonstrated the highest electrochemically active area of 0.267 cm^2^. Sol-gel process involves the formation of a gel from particles in a solution under controlled conditions. Due to the synergistic catalytic effect of the AuNRs and graphene, the modified electrode demonstrated accelerated electrocatalytic activity toward the oxidation of NO. The amperometric results showed a linear increase in current with increasing concentrations of NO within a range of 10–140 nM and a limit of detection of 6.5 nM.

A 3D graphene hydrogel (3DGH) has also been used to further enhance the electrochemical properties of graphene since its mechanically stable and porous 3D morphology provides exceptional conductivity, high surface area, and rapid electron mobility.^100^ Li *et al*.^101^ prepared an AuNPs embedded 3DGH via the *in situ* reduction of gold ions within the 3DGH matrix. Pristine 3DGH showed poor intrinsic electrocatalytic activity, while the uniform distribution of AuNPs on 3DGH efficiently and synergistically supported electron transport. A further advantage of 3DGH is the vacant inner matrix core with strongly shielded outer walls, which efficiently adsorbs and stores gases, thereby supporting the easy enclosure of NO for the effective *in situ* detection of NO secreted from living cells.

The doping of sulfur within the graphene lattice is an emerging approach to improve the affinity of an electrode toward NO since the interactions between sulfur and graphene synergistically provide more active sites for NO adsorption and storage. In a study reported by Bhat *et al*.,^102^ graphene doped with sulfur and coated with AuNPs exhibited a linear range between 24.9 and 680.93 *μ*M with a detection limit of 9 nM.

### B Graphene—Chalcogenides/transition metal composites

**Fig. 3:**
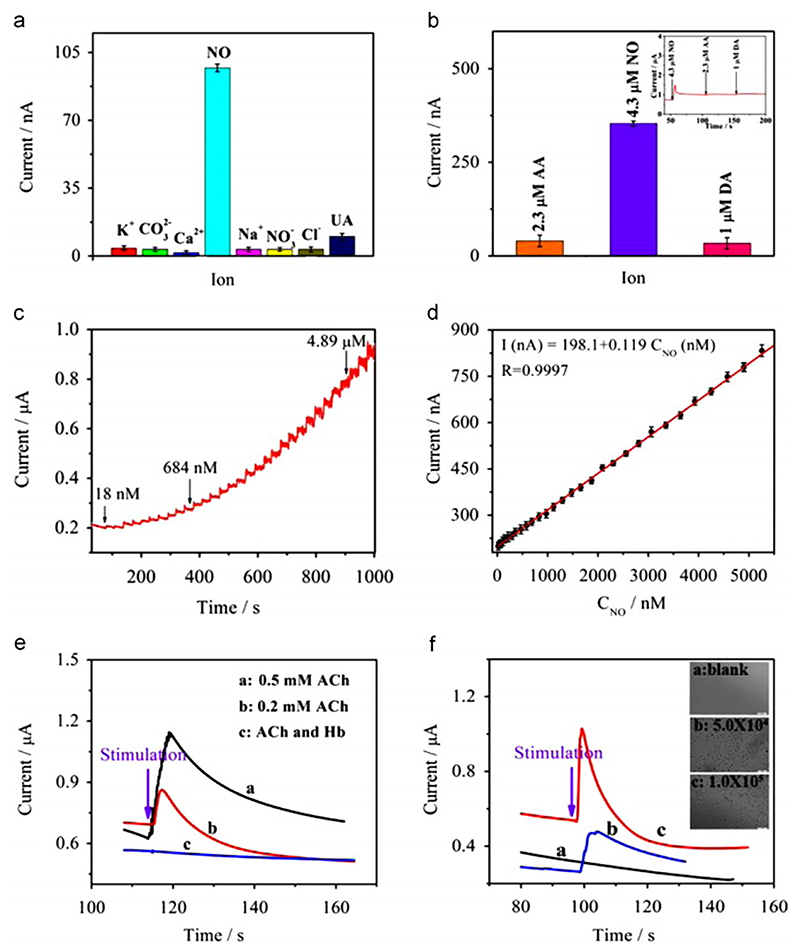
Electrochemical sensing performance of the graphene nanocomposite rGO–CeO_2_ composite/GCE for the detection of NO. (a) and (b) show specificity for NO even in the presence of potentially interfering biomolecules, ascorbic acid (AA), uric acid (UA), and dopamine (DA). (c) Amperometric response of the modified electrode with different NO concentrations at an applied potential of 0.8 V. (d) Calibration curve of modified rGO–CeO_2_/GCE electrode for NO, exhibiting a wide linear range of 18.0 nM to 5.6 *μ*M, a sensitivity of 1676.06 mA cm^−2^ M^−1^ and a low detection limit of 9.6 nM. (e) Real-time detection of NO secreted from cultured A549 cells stimulated by different amounts of acetylcholine (ACh) in cell culture medium with a cell density of 5 x 10^4^ ml^−1^ on rGO–CeO_2_ sensor. Ach is an endogenous neurotransmitter that stimulates NO release from endothelial cells, whereas hemoglobin (Hb) is a strong scavenger of NO and is used as a control. (f) Real-time detection of NO from A549 cells in cell culture medium with different cell densities. The insets are microscope photographs of A549 cells (n = 5). Reproduced with permission from Hu et al., Biosens. Bioelectron. **70**, 310–317 (2015). Copyright 2015 Elsevier.^107^

Alongside graphene, transition metals have also attracted attention as 2D materials with unique properties due to quantum size effect, size- and shape-dependent electrical, optical, chemical, and mechanical properties as well as surface chemistry.^103–105^ Among the transition metal oxides, cobalt oxide (Co_3_O_4_) has been noted as an excellent catalyst to fulfill the primary demand of electrode materials in terms of high catalytic activities and strong durability at a high range of applied potentials. For example, Lim *et al*.^106^ reported the development of an NO sensing platform based on Pt-modified Co_3_O_4_ doped rGO nanocomposite (rGO–Co_3_O_4_@Pt). A GO dispersion was treated with cobalt acetate and ammonia under ultrasonication to generate a homogeneous rGO–Co_3_O_4_ solution, followed by a hydrothermal reaction with PtNPs precursors to make rGO–Co_3_O_4_@Pt nanocomposite. Ammonia facilitated both the precipitation of the Co^2+^ ions and the reduction of GO. Interfacial Co–O–C bonds helped the strong anchoring of Co_3_O_4_ nanocubes with sp^2^ carbon of rGO. The electron-rich Co_3_O_4_ nanocenters allow the growth of PtNPs on their surface and corresponding Co_3_O_4_@Pt assembly behaves as spacers between discreet rGO sheets. Such arrangement acts as active sites for electron tunneling; thus, it was observed that the rGO–Co_3_O_4_@Pt nanocomposite-modified electrode showed sharp reversibility and an amperometric sensitivity of 0.026 ± 0.0002 *μ*A/*μ*M^−1^ with a limit of detection of 1.73 *μ*M for the electrochemical detection of NO. The rGO–Co_3_O_4_@Pt nanocomposite functionalized electrode transferred the oxidation overpotential of NO to a less positive potential in comparison to the other functionalized electrodes. Enhancement in electron-transfer kinetics results in a decrease in the potential, resulting in the oxidation of NO (Fig. 3).

Recently, cerium oxide (CeO_2_) NPs for the selective electrochemical oxidation of NO have also shown promise.^108,109^ Hu *et al*.^107^ fabricated rGO–CeO_2_ nanocomposites by adding variable ratios of cerous nitrate and ammonia to GO dispersions to obtain homogeneous rGO–CeO_2_ solution by undergoing initial nucleation of CeO_2_ followed by growth on rGO sheets. They observed the shape-dependent electrocatalytic activity of this composite, whereby the hexagonal nanocrystals exhibited a high negative oxidation potential due to a greater number of active sites and large surface area to volume ratio. This facilitated cell adhesion and demonstrated effective detection of cellular NO release. Deng *et al*.^110^ reported the facile fabrication of rGO doped with antimony tetroxide (Sb_2_O_4_) as an efficient electrochemical NO sensor. The presence of biphasic structure in Sb_2_O_4_, in combination with rGO is expected to act as a good electrocatalytic material for sensing. The detection limit was determined as 3.98 nM, with a linear detection range from 3.98 nM to 0.772 *μ*M.

Covalent organic frameworks (COFs) are porous organic networks linked by covalent bonds to form crystalline polymers, which allow the sophisticated incorporation of organic building blocks into an organized pattern.^111,112^ More recently, COFs have also shown promise as electrochemical sensing electrodes due to their highly exposed electrocatalytic architectures, and nitrogen-coordinated metal atoms. However, the mesoporous and microporous morphology of COFs has restricted their utility in electrochemical sensing. To address this challenge and to achieve the synergistic electrocatalytic effects, Zhu *et al*.^113^ reported the development of a covalent organic framework (COF-366) based electrochemical probe with 3D graphene hydrogel (3DGH) for NO sensing. The hierarchical porosity order of COFs often limits its use for catalysis; however, in combination with 3DGH surface and bulk matrix, it could be homogeneously placed over a large surface area, leading to significant electrocatalysis. Furthermore, the microporosity of 3DGH enhances the electron transfer and diffusion characteristics; together with the arrangement of nitrogen coordinated Fe–N–C active sites on graphene, it demonstrated enhanced electrocatalysis of NO with high sensitivity over a large range of 0.18 to 400 *μ*M as well as a low detection limit (30nM). In common with the above-mentioned GO-based sensors, this system also showed greater negative peak potential for NO, high reversibility of the redox processes with low peak-to-peak separation, and supported the real-time monitoring of NO released from HUVECs.

### C Graphene—Biomolecules composites

**Fig. 4:**
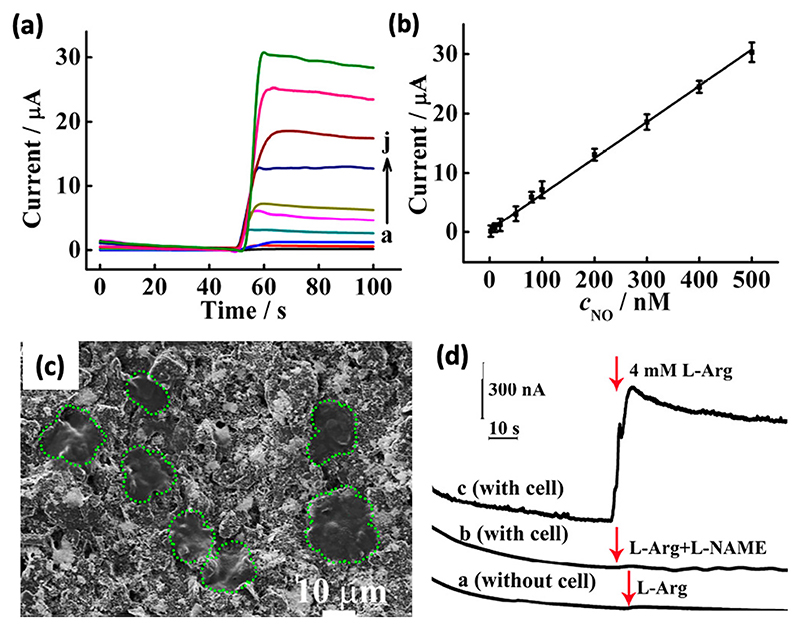
DNA-templated AuNPs on nitrogen-doped graphene sheets for electrochemical detection of NO. (a) Current–time responses of laminin/AuNPs-ctDNA-nitrogen doped graphene sheets (NGS)/screen-printed carbon electrode (SPCE) to 2,10, 20, 50, 80,100, 200, 300, 400, 500 nM of NO. (b) Calibration curve of the amperometric response vs the concentration of NO from 2 to 500 nM (*n* = 3). (c) Scanning electron microscopy image of MCF-7 cancer cells cultured on laminin/AuNPs-ctDNA-NGS/SPCE. (d) Current–time response curves of laminin/AuNPs-ctDNA-NGS/SPCE to (a) the addition of 4 mM L-Arg in the absence of cells, (b) the addition of 4 mM L-Arg and 4 mM L-NAME in the presence of cultured MCF-7 cells. A significant current response was only observed when 4 mM L-Arg was added to cultured MCF-7 cells (c). The red arrows indicated the injection of the drugs. L-Arginine (L-Arg) was used as a donor to generate NO, and L-NAME was used to inhibit the generation of NO. Reproduced with permission from Dou *et al*., Anal. Chem. **91**, 2273–2278 (2019). Copyright 2019 American Chemical Society.^114^

Although most of the graphene-based composites exhibit good electrocatalytic properties due to the electron-rich sites, modifying such systems with biomolecules such as DNA, RNA, and aptamers could be another strategy to improve real-time signal amplification and target-recognition, providing specificity by allowing precise control over NO recognition. Duo *et al*.^114^ demonstrated the *in situ* synthesis of highly catalytic DNA-templated AuNPs decorated on nitrogen-doped graphene sheets for electrochemical detection of NO secreted from live cancer cells. Each nucleotide of DNA is comprised of nitrogenated nucleobase, deoxyribose, and phosphate.^115^ The presence of such excessive binding sites has been used to prepare metallic NPs for the selective and sensitive detection of biomolecules of interest.^116,117^ DNA is reacted with HAuCl_4_ to produce a DNA–Au(III) complex, which is then chemically reduced.^118^ The nitrogen-doped graphene nanosheets show promising electrocatalytic activity for the oxidation of NO, corresponding to a conjugated system with the sp^2^-hybridized carbon frameworks fashioned by the sole electron pairs of nitrogen atoms. Further, the nitrogen-doping also provided adsorption sites for DNA binding as compared to pristine graphene nanosheets. In order to enhance the biocompatibility for culturing live cells on the surface, laminin was introduced on the nanocomposite. This nano-composite exhibited ultrasensitive and selective sensing of NO within the dynamic range of 2–500 nM with a detection limit of 0.8 nM. As illustrated in Fig. 4, the amperometric current response rises in a linear manner with increasing NO concentration.

NO exerts its physiological effects by direct binding of NO to ferrous and ferric hemes [explained earlier in (Eq. 1)]; this chemistry has been exploited for the specific sensing of NO by the chemical modification of the electrode surface with heme-containing proteins [e.g., hemoglobin (Hb), myoglobin (Mb), cytochrome c (cyt c), and soluble guanylate cyclase (sGC)]. Hemoglobin (Hb) is a vital metalloprotein found in red blood cells, where it functions as an oxygen carrier. Hb exhibits an excellent enzyme-like catalytic activity for the reduction of NO.^13,39^ In order to fully exploit the potential of Hb for the detection of NO, Hb has been conjugated with nanomaterials. Wen *et al*.^39^ took advantage of multiple biomolecules, including Hb, chitosan and hexadecyltrimethylammonium bromide (CTAB), for the development of a novel amperometric biosensing platform by immobilizing them along with graphene on a GCE to quantitatively measure NO. In this scenario, Hb was used as a nitrite reductase to generate NO; CTAB was used as a surfactant to reduce NP aggregation, and chitosan is a natural polymer that carries a primary amine group used for the purpose of conjugation in this study. This graphene-polymer-based sensor displayed superior performance compared to biosensing platforms prepared using carbon nanotubes, graphite, or GR/ionic liquid with a low detection limit of 6.75 nM (S/N = 3) and a high sensitivity of 615 *μ*A mM^−1^. Moreover, it exhibited excellent selectivity, reproducibility, and long-term storage stability, with a great potential for NO detection applications.

**Fig. 5:**
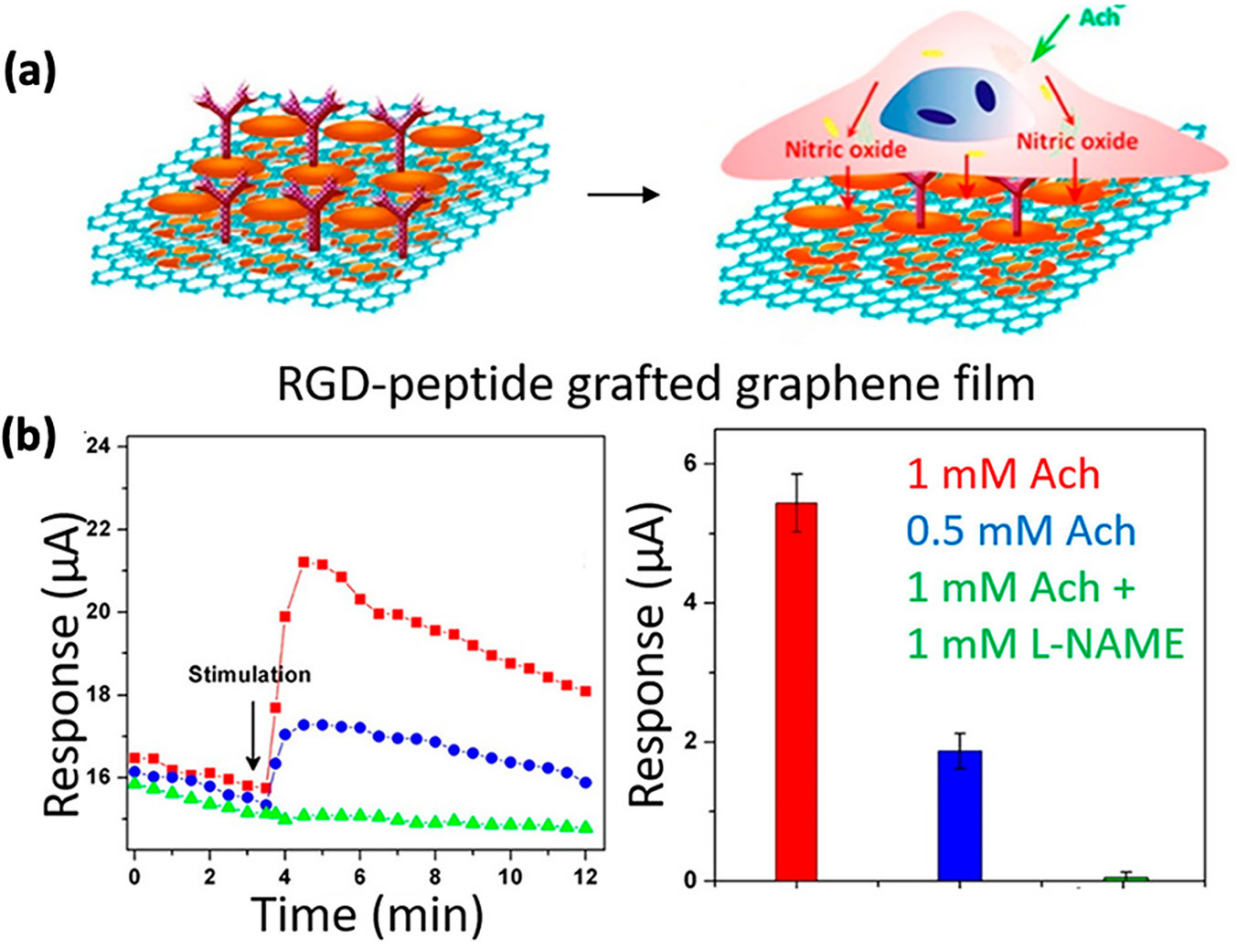
RGD-peptide functionalized graphene film for real-time detection of NO in HUVECs. (a) Illustration of RGD-peptide functionalized graphene film with HUVECs. (b) Real-time detection of NO released from HUVECs when treated with 1 mM acetylcholine to stimulate NO release (red), 0.5 mM acetylcholine (blue), and 1 mM acetylcholine with 1 mM L-NAME (green) demonstrating concentration changes and NO specificity when treated with a NO inhibitor (L-NAME). Reproduced with permission from Guo *et al*., ACS Nano **6**, 6944–6951 (2012). Copyright 2012 American Chemical Society.^121^

Other biomolecules such as myoglobin, arginine-glycine-aspartic acid (RGD)-peptide, Cyt c have also been used to conjugate or immobilize nanomaterials onto graphene to enhance the sensitivity and selectivity for NO. Marlinda *et al*.^119^ used a nanohybrid of gold nanorods, myoglobin, and reduced graphene oxide (rGO) to modify a GCE for the sensitive detection of NO. Similarly, Yoon *et al*.^120^ used a nanohybrid of Mb, graphene oxide (GO) and amine-modified molybdenum disulfide NPs (MoS_2_) to prepare an electrochemical biosensor for accurate detection of NO. Next, Guo *et al*.^121^ established a novel methodology to fabricate a free-standing biosensing platform using pyrenebutyric acid functionalized graphene film covalently decorated with RGD-peptide; they showed that the sensor measured NO released from HUVECs that were stimulated with 1 mM ACh; a limit of detection of 90 nM was demonstrated (Fig. 5). In this context, RGD-peptide was used to achieve cell attachment and growth on the graphene film surface, which in turn can allow sensitive and real-time detection of NO.

Chen *et al*.^122^ prepared a nanocomposite containing poly (1-vinyl-3-ethyl imidazolium) bromide, polymerized ionic liquid (PIL), and modified graphene nanosheet (PIL-Gr) as a modifier on the basal plane graphite (BPG) electrode surface for direct electrochemistry of Cyt c and NO sensing. Cyt c also contains heme groups, which provide exceptional electrocatalytic activity for the reduction of NO. The assembled sensor displayed a rapid response, good sensing, stability, and reproducibility for NO with an equivalent linear range and lower detection limit. They observed that the presence of Cyt c offered good electrochemical activity on the surface of the electrode and exhibited an enzyme-like activity for the reduction of NO. Such dual functionality opens up a new window to develop novel NO biosensors.

### D Other graphene composites

**Fig. 6:**
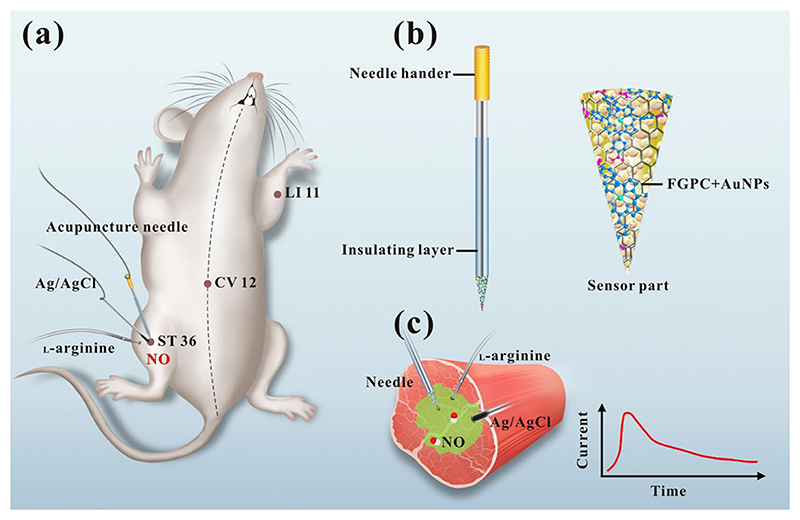
Schematic diagram of (a) a sensitive acupuncture needle microsensor for real-time monitoring of NO in acupoints of rats. (b) FGPC/AuNPs/acupuncture needle and (c) real-time NO measurement in acupoint ST 36 stimulated by L-arginine in the presence of NO synthase (NOS) under physiological conditions to produce NO. The functionalized needle is used for real-time detection of NO for different acupoints of rats, identifying *in vivo* NO detection. Reproduced with permission from Tang *et al*., Sci. Rep. **7**, 6446 (2017). Copyright 2017 Nature Springer.^123^

A sensing platform consisting of a graphene-modified acupuncture needle on the GCE surface has recently been described for *in vivo* biological applications by Tang *et al*.^123^ They used iron-porphyrin functionalized graphene (FGPC) to modify an acupuncture needle for the real-time detection of NO secreted in the acupoints of rats. Iron porphyrins were chosen to mimic the properties of heme proteins, such as hemoglobin and myoglobin. For this, hydrothermal synthesized FGPC was electrochemically deposited onto the surface of a gold-coated acupuncture needle. As illustrated in Fig. 6, AuNPs were sprayed onto the acupuncture needle by sputter coating. Iron-porphyrin functionalized graphene was deposited on the surface by the electrochemical deposition method. The modified needle can be applied to detect NO with high sensitivity and selectivity. They observed that the excellent conductivity of graphene and favorable catalytic features of iron-porphyrin enabled the detection of NO with high specificity and sensitivity at *in vivo* accupoints in rats with a lower detection limit of 3.2 nM. They contend that such a needle-based device could also be employed for detecting other important signaling molecules *in vivo* (e.g., hydrogen sulfide and carbon monoxide).

Ng and co-workers^124^ developed a nanocomposite gel that exhibited a uniform porous structure, which was synthesized by mixing an ionic liquid, 1-butyl-3-methylimidazolium hexafluorophosphate, with three-dimensional graphene material. The nanocomposite gel was cast on the GCE and covered with a Nafion^®^ layer. Under optimal experimental conditions, the developed sensing electrode exhibited a large dynamic response range and excellent stability with a high sensitivity of 11.2 *μ*A cm^−2^ (*μ*mol l)^−1^ and a low detection limit of 16 nM.

The results confirmed that such a nanocomposite gel can be used for sensitive NO detection.

Liu *et al*.^125^ developed a novel metalloporphyrin and 3-aminophenylboronic acid (APBA) co-functionalized reduced graphene oxide (rGO) based electrochemical sensing platform for NO. The assembly of highly catalytic metalloporphyrin with highly electric conductive rGO provides the sensor with sub-nM sensitivity. APBA is used as a cell-adhesive molecule to boost the biocompatibility of the sensing platform without reducing sensitivity; the reversible interaction between carbohydrates in the cell membrane permits the practical reusability of the sensor. Furthermore, the sensor was utilized for real-time sensing of NO in a human endothelial cell culture.

## VI Conclusions

The low concentration and high reactivity of NO in living cells and tissues have created a demand for highly sensitive and specific detection methods. While commercially available electrochemical NO sensors have proven to be useful research tools, there are opportunities for further optimization in terms of improved sensitivity, selectivity, and temporal resolution for real-time measurements under physiologically relevant conditions. Practical considerations also require optimization, for example, to reduce lengthy electrode surface polarization protocols, enhance mechanical flexibility, as well as improve the stability and degradation of the sensor.

In this review, we have made the case for functionalized graphene as part of a new generation of electrochemical NO sensors, thanks to its electrical conductivity, chemical properties, and high specific surface area. Most notably, graphene is easy to functionalize by combining different features of more than one material to achieve highly desirable electrode and electrocatalytic properties. This is a dynamic area of research, and we provide multiple examples of novel graphene nanocomposites, where the realization of these technological advances will ultimately require commercialization. In particular, the miniaturization of graphene-based sensors using a needle-based approach shows great promise for the real-time monitoring of NO in organs and tissues of interest.

Future work should focus on (i) preparation of graphene nanocomposites that are stable, biocompatible, and exhibit excellent corrosion resistance in different buffers, (ii) miniaturization of electrode systems with simple, facile, cost-effective, and straightforward preparation strategies that can avoid sensor degradation under harsh conditions, and (iii) use of these functionalized electrodes for the *in vivo* monitoring of NO. In this way, graphene-based electrochemical sensing has the potential to detect NO in an efficient, selective, and sensitive manner to significantly advance our understanding of fundamental biology.

## Figures and Tables

**Table I:** Advantages and limitations of commonly used methods for the detection of NO.^21–28^

Method	Advantages	Limitations
Griess assay	- Simple, fast and inexpensive colorimetric detection	- Limit of detection (0.5–2 *μ*M) higher than physiological range
	- Reaction is quantifiable by nitrite reference curve	- Sensitive to contamination by anticoagulants and proteins
	- Ready-to-use kits available	- No spatial information
Ozone-based chemiluminescence	- High sensitivity (~1 nM) and reproducibility	- Specificity issues, since it can also detect other NO_x_ species
	- Gold standard for quantification	- Frequent calibration
	- Can be used with cell lysates and tissue homogenates	- Proteins may cause foaming
	- Can be used to detect s-nitrosothiols by conversion to NO	
Fluorescence detection method	- Detection using microscope, plate reader or flow cytometer	- Requires aerobic conditions
		- Semiquantitative
	- Allows spatial and temporal imaging	- Reacts with oxidized NO metabolites, especially N_2_O_3_
	- Cell permeant version	- Interference from intracellular oxidants and antioxidants
	- Mostly in *vitro*, but intravital also possible	- Some probes sensitive to pH and Ca^2+^
	- Sensitivity: 5 nM	
Electron paramagnetic resonance (EPR) spectroscopy	- Highest specificity due to highly effective NO trap	- Ease of oxidation under aerobic conditions to form heme complexes and reactive oxygen species
	- Real-time monitoring of NO	
		- Expensive and requires specialist expertise
Clarke-type electrodes	- *In situ* data acquisition	- Electrode pretreatment steps
	- Real-time detection.	
	- Sensitivity: above 1 nM	- Electrode fouling
	- Ease of adjusting temperature, and solution pH.	- Electrode fragility
		- Frequent calibration

**Table II:** Summary of electrochemical detection of NO by graphene nanocomposites.

Electrode composition	Linear range (*μ*M)	Sensitivity	Limit of detection (nM)	Working potential	Test sample	Reference
Graphene paper that was loaded with Au@Pt core–shell nanoparticles (NPs)	400 nM to 673.9 μM	3.653 mA cm^−2^ mM^−1^	100 nM	0.75 V	Human umbilical vein endothelial cells (HUVECs)	93
Reduced graphene oxide (rGO) and AuPt bimetallic NPs based composite	0.02–1.8 *μ*M	7.35 *μ*A *μ*M ^−1^	2.88 nM	−1.5 V	Rat cardiac cells (H9C2 cells; ATCC)	94
AuNPs conjugated sulfur-doped graphene nanohybrids	24.9–680.93 μM	21046.72 *μ*A M^−1^ cm^−2^	0.009 *μ*M	−1.2 V	Phosphate buffered saline (PBS)	102
Hybrid film of an electrochemically reduced GO conjugated with AuNPs	Up to ~3.38 *μ*M	5.38 *μ*A/*μ*M/cm^2^	133 nM	0.821 V	HUVECs	95
rGO and Nafion^®^ (Nf) based film loaded with AuNPs	1 *μ*M to 0.16 mM	Not given	0.5 *μ*M	0.8 V	PBS	96
Amine functionalized silicate matrix loaded with rGO and Au nanorods	10 to 140 nM	0.598 nA/nM	6.5 nM	0.8 V	PBS	99
AuNPs embedded 3D graphene hydrogel	0.2 to 6 *μ*M	Not given	9 nM	0.81 V	Normal murine skin cells JB6-C30 and tumor cells B16-F10	101
AuNPs conjugated with graphene	10–5000 *μ*M	0.1212 *μ*A*/μ*M^−1^	0.04 *μ*M	0.8 V	PBS	126
Pt NPs-decorated electrochemically reduced GO GCE	0.25–40 *μ*M	8.40 *μ*A *μ*M^−1^ cm^−2^	52 nM	0.5 V	Human serum sample	127
rGO–cobalt oxide nanocube@Pt (rGO–Co_3_O_4_@Pt) nanocomposite	10–650 *μ*M	0.026 ± 0.0002 *μ*A *μ*M^−1^	1.73 *μ*M	0.87 V	PBS	106
rGO conjugated with ceria (rGO–CeO_2_)	18.0 nM to 5.6 *μ*M	1676.06 mA cm ^−2^ M^−1^	9.6 nM	0.8 V	Human lung carcinoma cells (A549)	107
Antimony tetroxide (Sb_2_O_4_) nanoflowers conjugated with rGO nanocomposite	3.98 nM to 0.772 *μ*M	74.59 *μ*A cm ^-2^ *μ*M ^-1^	3.98 nM	0.84 V	HUVEC and tumor cells A549	110
Covalent organic framework (COF)-366-Fe on 3D graphene aerogel	0.18 to 400 *μ*M	8.8 *μ*A·*μ*M^−1^·cm^−2^	30 nM	0.53 V	HUVECs	113
Porphyrin functionalized nitrogen doped graphene nanosheets	10nM–10 *μ*M	3.6191 *μ*A *μ*M^−1^	1nM	−0.6 V	Murine macrophage RAW 264.7 cell	128
Zinc-dithiooxamide framework derived porous ZnO NPs that were conjugated with polyterthiophene–rGO composite	0.019–76 × 10^−6^M	0.019–76 × 10^−6^M	7.7 ± 0.43 × 10^−9^M	−0.81 V	Cancerous PC-12 and the normal HEK-293	129
Iron phthalocyanine (FePc) functionalized nitrogen-doped graphene composite	1.8 × 10^−7^ to 4 × 10^−4^mol l^−1^	0.21 *μ*A *μ*M^−1^ cm^−2^	180 nmol l^−1^	0.9 V	HUVECs	130
DNA-templated AuNPs decorated on the nitrogen-doped graphene sheets	2−500 nM	860.9 *μ*A *μ*M^−1^ cm^−2^	0.8 nM	0.8 V	MCF-7 cells	114
Hemoglobin immobilized on graphene electrode modified by chitosan and hexade-cyltrimethylammonium bromide (CTAB)	9.0 × 10^−8^ to 2.5 × 10^−6^M	316 *μ*A mM^−1^	6.75 × 10^−9^M	−0.70 V	PBS	39
Myoglobin-modified Au nanorods conjugated with rGO	10 to 1000 *μ*M	0.0539 *μ*A/*μ*M^−1^	5.5 *μ*M	0.85 V	PBS	119
Nanohybrid of myoglobin, GO and amine-modified molybdenum disulfide (MoS_2_) NP	Not given	5 × 10^−7^ (A/V)	3.6 nM	−0.3 V	PBS	120
Pyrenebutyric acid functionalized graphene film covalently decorated with arginine-glycineaspartic acid (RGD)-peptide	Not given	Not given	25 nM	0.75 V	HUVECs	121
Cytochrome c (Cyt c) immobilized polymerized ionic liquid (PIL), poly (1-vinyl-3-ethyl imidazolium) bromide, modified graphene	1.05 × 10^−6^ to 1.37 × 10^−5^M	Not given	7.0 × 10^−7^M	−0.73 V	PBS	122
Acupuncture needle modified with an iron-porphyrin functionalized graphene	5 nM to 200 nM	Not given	3.2 nM	+0.78V	*In vitro:* HUVECs; *in vivo:* male rat subcutaneously at acupoints	123
Silver NPs-supported rGO	10–220 *μ*M	0.01065 *μ*A *μ*M^−1^	2.84 *μM*	+0.96 V	PBS	58
Nanocomposite of graphene, Nafion^®^ and AuNPs	36 nM to 20 *μM*	0.0275 + 0.0703 *μ*A *μ*M^−1^	18 nM	0.8 V	Fish liver homogenate stimulated by L-arginine	97
3D graphene material with an ionic liquid, 1-butyl-3-methylimida-zolium hexafluorophosphate	up to 16 *μM*	11.2 *μ*A cm^−2^ (*μ*mol l^−1^)^−1^	16 nM	Not given	PBS	124
Metalloporphyrin and 3-aminophenylboronic acid (APBA) co-functionalized rGO	Not given	37.6 *μ*A *μ*M^−1^ cm^−2^	55 pM in PBS and 90 pM in a cell medium	+0.75 V	HUVECs	125

## Data Availability

The data that support the findings of this study are available within the article.
